# Investigation of Phase‐Change Droplets and Fast Imaging for Indicator Dilution Measurement of Flow

**DOI:** 10.1002/jum.16722

**Published:** 2025-05-19

**Authors:** Zachary Zajac, Brandon Helfield, Ross Williams, Paul Sheeran, Charles Tremblay‐Darveau, Kimoon Yoo, Peter N. Burns

**Affiliations:** ^1^ Department of Medical Biophysics University of Toronto Toronto Ontario Canada; ^2^ Physical Sciences Department Sunnybrook Research Institute Toronto Ontario Canada; ^3^ Department of Physics Concordia University Montreal Quebec Canada; ^4^ Department of Biology Concordia University Montreal Quebec Canada

**Keywords:** droplets, indicator dilution, ultrasound contrast agents

## Abstract

**Objectives:**

The development of low boiling point liquid droplets as phase‐change contrast agents allows for the local creation of microbubbles at a point of interest in vivo. Although there are many possible applications, few investigations have used selectively created microbubble boluses to measure volumetric flowrate. In this study, the flow ratio between two vessels is calculated by vaporizing droplets in each vessel individually.

**Methods:**

Proof of principle is demonstrated in vitro by an imaging sequence that vaporizes droplets using a high mechanical index pulse, then images the transit of the resulting microbubbles at a high frame rate using low mechanical index plane waves.

**Results:**

It is shown that a linear relationship exists between the concentration of droplets and enhancement of the resulting microbubble bolus. In vitro flow is measured with a mean error of 8% in a 0.66 cm diameter vessel and with a mean error of 33% in a 0.49 cm diameter vessel. The relative volumetric flow between two adjacent vessels is calculated with a mean percentage error of 25% when imaging the region of droplet vaporization for flow ratios between 0.25 and 4.

**Conclusions:**

This in vitro study demonstrates the feasibility of using a positive bolus tracer, induced by image‐guided ultrasound excitation, to measure flow. Potential applications include measurement of the portal vein to hepatic artery flow ratio, known as the hepatic perfusion index.

**Abbreviations**
DFB: decafluorobutane
HPI: hepatic perfusion index
MI: mechanical index
PRF: pulse repetition frequency
ROI: region of interest

Microbubble contrast agents have provided a method to measure volumetric blood flowrate in small vessels. While infusing the bubbles so that a steady concentration is attained in blood, they can be disrupted in a selected imaging plane using one or two frames of high mechanical index (MI) sound. Subsequently, the rate at which new bubbles replenish the plane is monitored by low MI imaging. A series of models have been developed that relate this time‐replenishment curve to relative flowrate, as well as to relative vascular volume and vessel geometry.^1^, ^2^ This technique can be thought of as a “negative bolus” indicator dilution method and has the advantage that it does not rely on the estimation of an unknown input function of a bolus of tracer injected into the circulation. Among its limitations, however, is that it cannot be used to measure flow from a single vessel that is adjacent to another within the effective volume of the disrupting beam if the replenishment cannot be resolved between the two vessels, as both will contain equal concentrations of bubbles. Such is the case in the hepatic artery and portal vein, for example, whose branches run close to each other near and within the liver. As both are infused with agent during the steady state, only their combined flow can be measured with this method. Yet the ratio of portal venous to hepatic arterial flowrate, the so‐called hepatic perfusion index (HPI),^3^ is a quantity that would be useful to measure with ultrasound.^4^

In this paper, we demonstrate the principle of a method with potential to achieve this using phase change sub‐micron droplets, which on activation by ultrasound, create a bolus of microbubbles that is localized in space and time. By injecting a bolus of droplets and performing the activation first in the arterial phase, when bubbles will be created only in the hepatic artery, and then in the portal venous phase, when bubbles will be created in both vessels, the ratio of two flows can be measured. Here, we demonstrate the feasibility of this concept by a series of in vitro experiments.

## Phase‐Change Droplets as a Contrast Agent

Low boiling point phase‐change contrast agents are sub‐micron droplets of a liquid precursor of microbubbles, typically a perfluorocarbon such as C_4_F_10_.^5^ Liquid perfluorocarbons are chosen because in addition to their low boiling point, they have low toxicity and low solubility in water.^6^ While such droplets cannot typically be visualized on ultrasound imaging, upon insonation above a specified peak negative pressure, the liquid perfluorocarbon core will vaporize.^7^ A gas pocket within the droplet grows, converting the droplet to a visible microbubble about 5–10 times larger in diameter than the droplet.^8^ The resulting microbubble can be detected using established contrast specific imaging methods.^9^, ^10^ Synthesis of sub‐micron droplets can be achieved by condensing existing microbubbles,^11^ sonicating droplet constituents,^12^ or microfluidic processing.^13^ Droplets have a circulatory half‐life of up to 60 minutes; an order of magnitude longer than that of a microbubble contrast agent^14^ and may be vaporized using exposure parameters that lie within current guidelines for diagnostic imaging.^15^ The formulation and synthesis of the droplets determine their size, stability as a droplet, stability as a microbubble, and the pressure required for their vaporization.^15^

By creating a localized bolus of bubbles following insonation of a chosen region of interest (ROI) in a vessel carrying droplets, the volumetric flowrate can be deduced by measuring the rate at which the bubbles leave the ROI as they are carried downstream. Here we demonstrate that, in principle, this can be used to determine the ratio of flowrates between two vessels that both lie within the region of interest.

## Indicator Dilution Flow Measurement

When a tracer is injected into the circulatory system and its intensity measured at distinct locations, tracer concentration may be inferred, allowing for hemodynamic properties to be estimated.^16^ This technique, known as indicator dilution, relies on assumptions which include mass‐conservation and perfect mixing, instantaneous injection, and constant flow.^17^ However, these assumptions are often violated in practice. For example, contrast agents may decay over time^18^ and recirculate.^19^ Modeling can help to alleviate these issues: curve fitting models may be applied to time‐intensity curves to correct for recirculation and to filter noise,^20^ and deconvolution techniques can be used to preserve the assumption of instantaneous tracer injection.^21^ Indicator dilution has numerous applications in medicine, including measuring cardiac output,^22^ pulmonary blood flow,^23^ and detecting right‐to‐left cardiac shunts.^24^

Assuming a closed system where the only tracer present is that which is injected, the indicator is well mixed and the mass of the indicator is conserved over time, volumetric flow rate is given by:

(1) $$F=\frac{q}{\int_{0}^{\infty }c\left(t\right)\mathrm{dt}}$$

where F is the volumetric flowrate of the system, q is the mass of tracer injected and $c\left(t\right)$ is tracer concentration as a function of time. This is known as the Stewart‐Hamilton relation.^25^, ^26^

A variation of the Stewart‐Hamilton relation provides a method to measure flow even if the mass of tracer—such as a microbubble bolus—within a region is unknown. If we define $q_{0}$ as the initial mass of indicator which is produced and *h*(*t*) as the fraction of tracer that exits a fixed region surrounding the tracer in time *dt*, we may write:

(2) $$\int_{0}^{\infty }q\left(t\right)\;\mathrm{dt}=q_{0}\;\int_{0}^{\infty }\left(1-h\left(t\right)\right)\;\mathrm{dt}$$

(3) $$q_{0}\bar{t}=q_{0}\int_{0}^{\infty }\left(1-h\left(t\right)\right)\;\mathrm{dt}$$

where $\bar{t}$ is defined as the mean transit time. Therefore:

(4) VF=∫0∞1−htdt

where *V* is the 3‐dimensional volume of the ROI drawn around the microbubble bolus. We may now write:

(5) $$F=\frac{V}{\int_{0}^{\infty }\left(1-h\left(t\right)\right)\;\mathrm{dt}}$$

Assuming a linear relationship between contrast density and echogenicity, this equation allows us to measure the volumetric flow rate without knowledge of the precise mass of bubbles that are produced from droplet vaporization. Instead, we can measure the fraction of bubbles that are remaining within an area of interest over time. By dividing the volume of the area of interest over time by the integral of this curve, known as the residual function, we can obtain the volumetric flow rate of the entire system.

(6) $$F=\frac{V}{\int_{0}^{\infty }R\left(t\right)\mathrm{dt}}$$

where *R*(*t*) is the fraction of tracer remaining within the ROI as a function of time.

Equation (6) is used in this paper to calculate the volumetric flow rate within each vessel. The volume of the ROI is calculated as the 2‐dimensional area of the ROI multiplied by the elevational beam width at the depth of imaging. The residual curves were calculated from the time course of the mean echo intensity within the ROI, measured from the ultrasound images.

## Materials and Methods

### Droplet Synthesis

Sub‐micron droplets were prepared according to a previously described method.^27^ Microbubbles comprising a C_4_F_10_ (decafluorobutane [DFB]) core (boiling point = −2.1°C^28^) and DBPC/DPPE‐PEG5k lipid mixture for the shell were condensed into droplets by manipulation of temperature and pressure. The peak diameter of the DFB DBPC/DPPE‐PEG5k microbubbles was 820 ± 10 nm and the concentration was 2.1 ± 0.5 × 10^9^ bubbles/mL, measured prior to condensation using a Coulter counter (Multisizer 3, Beckman Coulter, Brea, CA, USA). Note that as the Coulter counter was unable to resolve particles smaller than 800 nm, a larger number of droplets than bubbles were counted. After condensation, the droplet solution was characterized by a peak diameter of 114 nm and a concentration of 9.1 × 10^12^ droplets/mL,^29^ as measured using a Nanosight (LM10, Malvern Panalytical, Malvern, UK). Experiments were carried out using droplets diluted in de‐ionized water at 37°C.

### Flow Phantom

**Figure 1:**
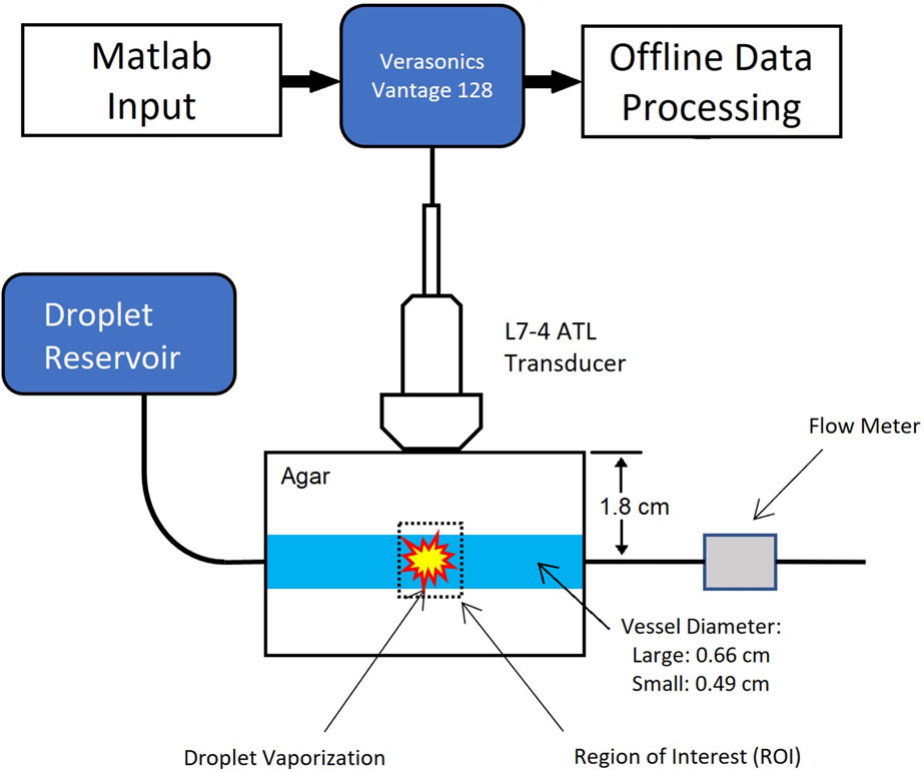
Schematic of experimental setup for the measurement of the time intensity curves following droplet vaporization. DFB (decafluorobutane)—DBPC/DPPE‐PEG5000 droplets were used at a concentration of 2.7 × 10^9^ droplets/mL.

A schematic of the flow phantom is shown in Figure 1. It comprised 4% agar containing a wall‐less 0.66 cm diameter vessel and a wall‐less 0.49 cm diameter vessel. These diameters were chosen as they are comparable in size to the hepatic portal vein and hepatic artery, respectively.^30^ Both vessels ran parallel to each other with their centers both at a depth of 1.8 cm. For every experiment, the flow phantom was placed into a 37°C water bath. A reservoir was employed to independently supply a given vessel with a dilute suspension of droplets maintained at 37°C, by gravity feed. The flowrate from the reservoir was adjusted by opening or closing downstream valves. Floating ball flowmeters (Cole‐Parmer, Vernon Hills, IL, USA and VWR, Radnor, PA, USA) were placed downstream of the flow phantom to provide a reference measurement of flowrate. The mean error given by the flow meter was −0.27% ± 3.10% (SD) calculated from 24 measurements ranging from flow rates of 38 to 316 mL/minute.

### Microbubble Imaging and Droplet Vaporization

A custom pulse sequence for droplet vaporization and microbubble imaging was developed on a Vantage 128 digital research platform (Verasonics, Kirkland, WA, USA) using an L7‐4 linear array transducer (ATL Ultrasound Inc., Bothell, WA, USA) with an elevational focus of 6 mm and a center frequency of 5.2 MHz. Vaporization of the droplets was accomplished with 4 two‐cycle focused pulses at a center frequency of 5.2 MHz, allowing for a clinically relevant MI and a pulse repetition frequency (PRF) of 2 kHz. A total of 128 continuous transducer elements were used for vaporization without aperture weighting. The focus was aligned with the center of the transducer and the axis of the vessel. The peak negative pressure for droplet vaporization measured with a hydrophone (Model 804, Sonora/Acertera, Longmont, CO, USA) was 2.62 MPa at a depth of 1.8 cm from the transducer, giving a MI of 1.15. The microbubbles were imaged with two‐cycle pulse‐inversion plane waves at a frequency of 3.5 MHz. This imaging sequence was initiated 10 ms after the vaporization sequence to allow the transducer to lower its voltage profile. These two pulse‐inverted plane waves were transmitted over 9 angles spaced evenly from −5 degrees to +5 degrees with a pulse repetition frequency of 5 kHz, which were then compounded to produce a single image. Fundamental mode imaging reconstructed the image through the summation of the 18 pulses, while contrast mode imaging reconstructed the image through the difference of the 18 pulses. The resulting image framerate was 116 frames/second. The peak negative pressure for imaging was measured at 488 kPa at a depth of 1.8 cm from the transducer, giving a MI of 0.26. The elevational beam width at the depth of imaging was measured as the full width at half maximum using the hydrophone. At a depth of 1.8 cm, the elevational beam width was 2.6 mm. Imaging data were offloaded for analysis using MATLAB (MathWorks, Natick, MA, USA).

### Relationship Between Droplet Concentration and Microbubble Bolus Intensity

In order to test the relationship between droplet concentration and intensity of the ensuing microbubble population after vaporization, droplets were diluted to estimated concentrations between 2 × 10^8^ and 1.6 × 10^10^ droplets/mL and flowed at 50 mL/minute within the 0.66 cm vessel. Data sequences before and after activation were stored and analyzed offline to characterize contrast enhancement as a function of droplet concentration and time after activation. The mean echo intensity inside an ROI surrounding the microbubbles was calculated for each of the 10 separate vaporization events at increasing droplet concentrations. Results were normalized with respect to the maximum average echo considering multiple bolus activations and are presented with the standard error of each measurement.

### Lifetime of Activated Droplets

To measure the lifetime of newly‐created DFB DBPC/DPPE‐PEG5k microbubbles, droplets were diluted to a concentration of 2.7 × 10^9^ droplets/mL and passed through the 0.66 cm diameter vessel. The flow was then stopped, and droplets were activated with a series of 30 focused pulses at an MI of 1.15, PRF of 2 kHz, and frequency 5.2 MHz, so as to create a large amount of contrast. The bubbles were then imaged with pulse‐inversion angle‐compounded plane waves at MI = 0.26, *f*_c_ = 3.5 MHz, and PRF = 5 kHz. The mean intensity within a stationary ROI enveloping the microbubbles was measured over time with fundamental mode imaging for 8 different activation events.

### Measurement of Flow in a Single Vessel

A suspension of droplets at concentration of 2.7 × 10^9^ droplets/mL was passed through each vessel of the phantom. After vaporization within the vessel, the normalized mean intensity inside the ROI was measured as a function of time as the tracer left the region, giving a residual curve *R*(*t*). The flowrate was calculated using Equation 6. This was calculated for 5 separate vaporization events. Flowrates of zero, 40, 60, 80, 100, 120, 140, and 160 mL/minute were tested. This was repeated for 4 different droplet samples. Both the large and small vessels were tested. The flowrate was also calculated for the stopped‐flow case, where there was no flow and the mean intensity within the ROI decreased due to decay, disruption, or diffusion of the newly created microbubbles.

### Measurement of Flow Ratio

The flow ratio between two vessels was estimated using the data obtained from the flow measurements in a single vessel. For each flow ratio calculated, the numerator was the mean flowrate of 20 bolus activations in the small vessel, while the denominator was the mean flowrate of 20 bolus activations in the large vessel. Flow ratios were calculated for every combination of flowrates between the large and small vessels. These measured flow ratios were then compared with the true flow ratio determined by the flowmeters.

## Results

### Relationship Between Droplet Concentration and Microbubble Bolus Intensity

**Figure 2:**
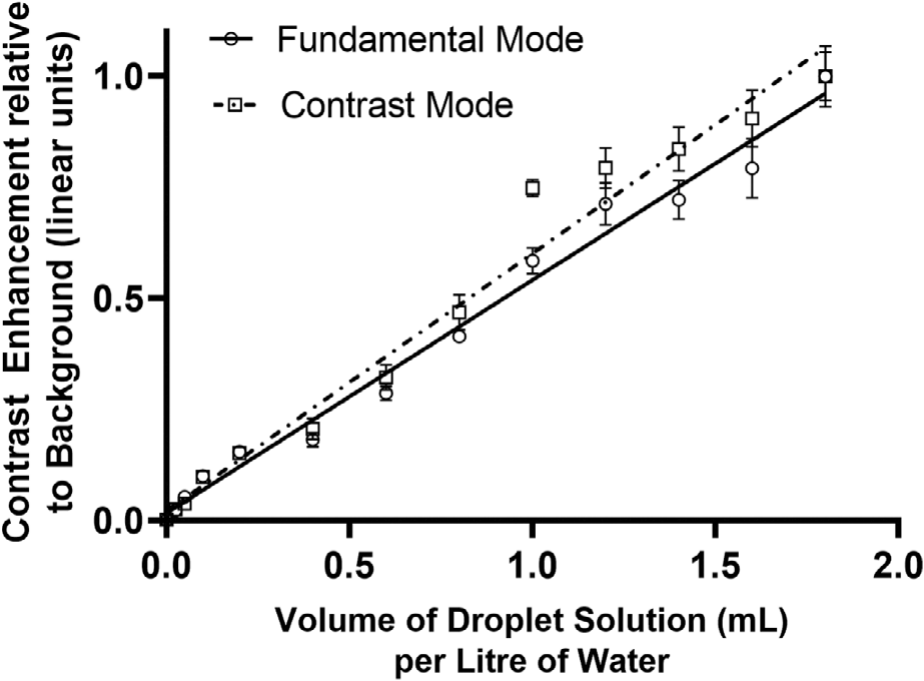
Mean intensity of microbubble bolus produced after vaporization versus droplet concentration viewed in fundamental mode (solid line) and in pulse inversion contrast mode (dash‐dotted line). The number concentration of the droplet solution was previously measured as 9.1 × 10^12^ droplets/mL.^29^ Imaging was performed at a frequency of 3.5 MHz and MI = 0.26. Error bars represent the standard error.

**Figure 3:**
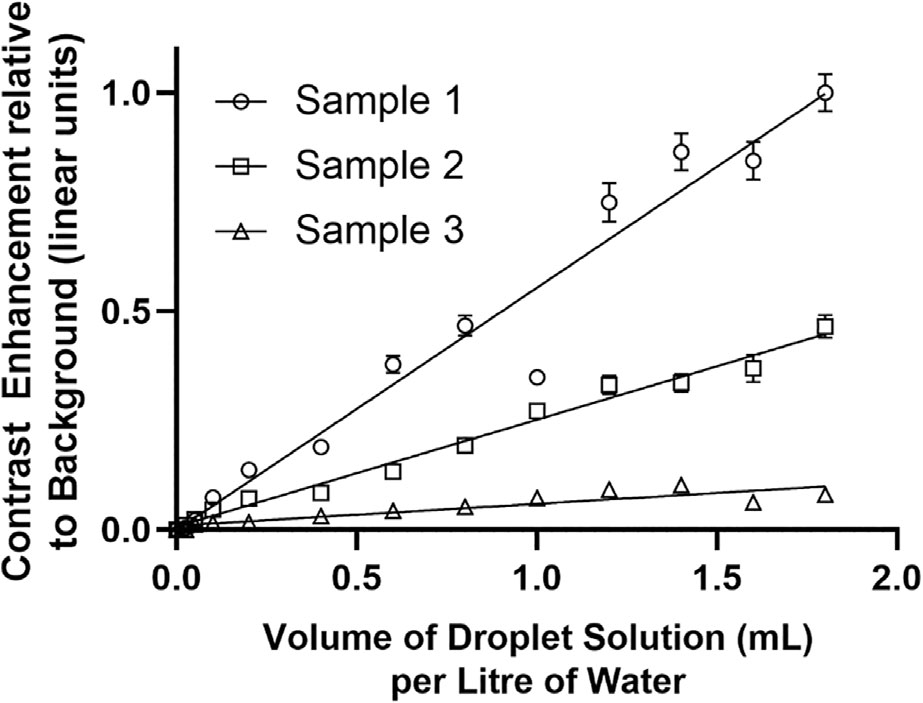
Mean intensity of microbubble bolus produced after vaporization versus droplet concentration showing linear enhancement but significant inter‐vial variability. The number concentration of the droplet solution was previously measured as 9.1 × 10^12^ droplets/mL.^29^ Imaging was performed at a frequency of 3.5 MHz and MI = 0.26. Error bars represent the standard error.

The maximum contrast enhancement observed after activation was 20.1 dB in fundamental mode and 28.5 dB in contrast mode, consistent with previous studies of nanoscale phase‐shift agents.^14^ Figure 2 shows a linear best‐fit relationship between droplet concentration and microbubble contrast enhancement in both fundamental and contrast mode imaging in a single vial, demonstrating the linearity of both methods. However, there was significant variation in absolute enhancement levels between the sample vials (Figure 3).

### Lifetime of Activated DFB DBPC/DPPE‐PEG5K Droplets

**Figure 4:**
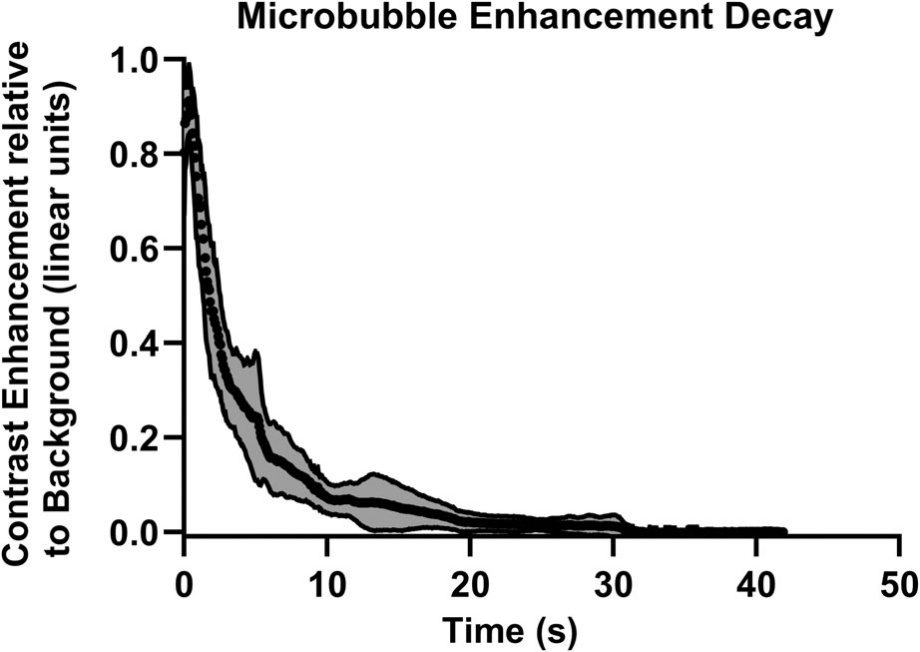
Mean intensity over time of a bolus of bubbles from newly activated DFB DBPC/DPPE‐PEG5000 droplets by fundamental imaging, mean and standard deviation of 15 trials. An exponential fit gives a half‐life of 2.5 seconds with *R* ^2^ = 0.98.

Figure 4 shows how the mean microbubble intensity in fundamental mode changed with time at no flow, averaged for 14 trials. The mean half‐life was 2.3 ± 0.4 seconds; an exponential fit gives a half‐life of 2.5 seconds with *R* ^2^ = 0.98. The error bars correspond to the standard deviation of the mean intensity within each ROI.

### Measurement of Flow in a Single Vessel

**Figure 5:**
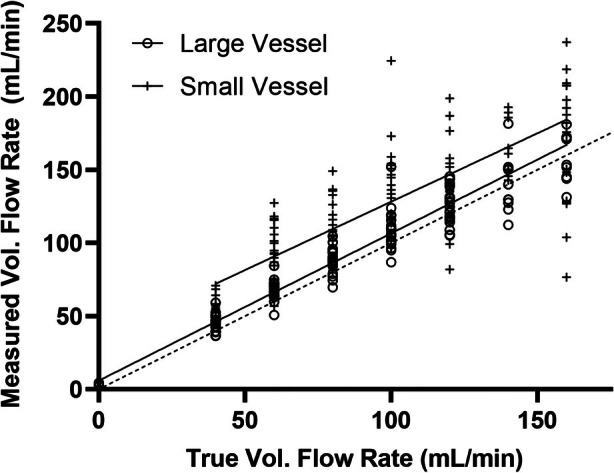
Flowrate measured using the intensity of residual bubbles from activated droplets versus true flowrate. The 0.66 cm diameter vessel (O) and 0.49 cm diameter vessel (+) are shown. The line of best fit for the larger vessel (solid line) is *y* = 1.01*x* + 5.90 with *R* ^2^ = 0.88 and 95% confidence intervals of (0.94, 1.07) and (−0.38, 12.18) for the slope and the y‐intercept, respectively; for the small vessel (solid line), *y* = 0.93*x* + 34.74 with *R* ^2^ = 0.48 and 95% confidence intervals of (0.76, 1.11) and (15.53, 53.95) for the slope and the y‐intercept, respectively. Error bars represent the standard error of the mean. The identity line (dashed) is shown for reference.

Once activated, the rapidity with which the bubbles left the region of interest required the use of high framerate planewave imaging; in all but the slowest flows, the bubbles had left the ROI completely within 200 ms, requiring framerates of greater than 100 Hz. At this frame rate of 116 Hz, approximately 23 bubble measurements are made before they leave the ROI. A greater rate may increase the risk of bubble disruption, inducing error in flow measurement. Figure 5 shows the results of the measurement of flow in each of the two vessels. The line of best fit from the large vessel was *y* = 1.01*x* + 5.90 with *R* ^2^ = 0.88, while the line of best fit for the smaller vessel was *y* = 0.93*x* + 34.74 with *R* ^2^ = 0.48. The mean percentage error of the flow calculation in the large vessel was 7.9%, and the mean coefficient of variation was 7.7%. For the small vessel, the mean percentage error of the calculated flow was 32.2% with a mean coefficient of variation of 5.0%.

### Determination of Flow Ratio

**Figure 6:**
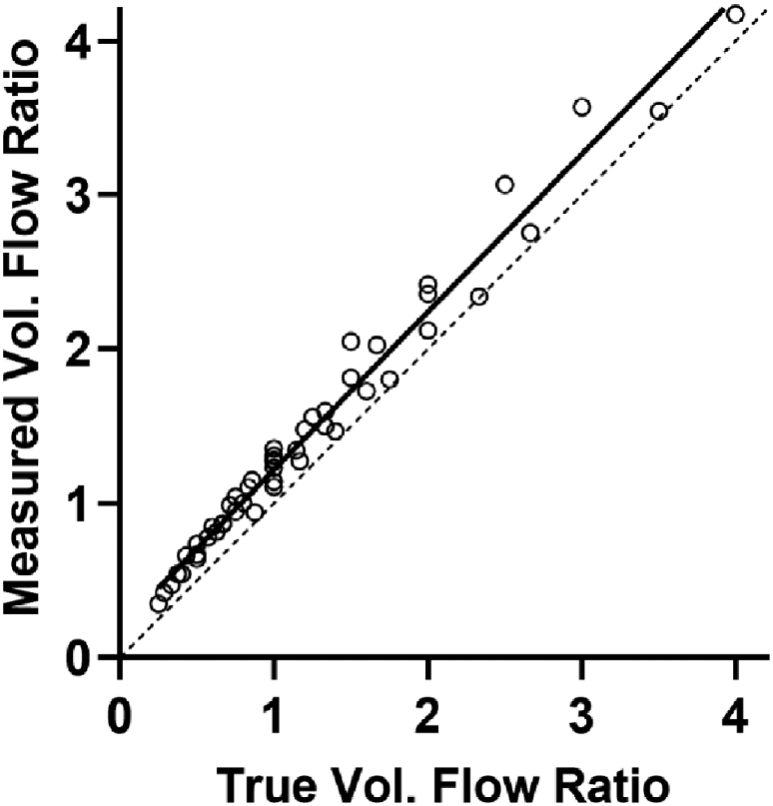
Flow ratio measured between two vessels using ROI intensity to determine the residual function. The identity line (dashed) is shown for reference. The mean and standard error of 5 measurements are shown. The line of best fit (solid line) gives *y* = 1.02*x* + 0.20 with *R* ^2^ = 0.98 and 95% confidence intervals of (0.98, 1.07) and (0.13, 0.26) for the slope and the y‐intercept, respectively. The mean percentage error was 24.7%.

The flow ratio between the two vessels was calculated from the time‐intensity curves. Using the mean intensity of the fundamental echo within the ROI as a measure of microbubble concentration, the line of best fit gives *y* = 1.02*x* + 0.20 with *R* ^2^ = 0.98 (Figure 6). The mean percentage error was 24.7%.

## Discussion

The combination of phase‐change submicron droplets and high‐speed planewave imaging presents a new opportunity for the measurement of flow without the need to tackle the established hurdles of measuring vessel diameter and mean velocity required by the more conventional 1‐dimensional pulsed Doppler approaches.^4^, ^31^, ^32^ These methods are particularly prone to measurement error from the Doppler angle and vessel cross‐sectional area,^33^, ^34^ making them especially difficult in small vessels such as the hepatic artery, whose diameter is typically about 3.5 mm.^35^ More recent implementations of the attenuation‐compensated flowmeter using a matrix array transducer^36^, ^37^ have overcome these limitations, providing angle‐independent measurement in which the vessel diameter is inferred from the Doppler signal intensity, but resolution at present limits the method to somewhat larger vessels. Combining Doppler with blood speckle decorrelation and high frame‐rate imaging, however, offers the potential to measure flow rate in small vessels.^38^ Such Doppler methods complement those made available by the advent of contrast agents and, of course, can be used in conjunction with them. The method proposed here is based on indicator dilution and shares with the negative bolus microbubble technique the advantage that a bolus input approaching a delta function is achieved with a minimally invasive method: in the microbubble technique by the disruption of bubbles in the ultrasound beam; in the present method by the creation of bubbles by the ultrasound beam. However, unlike the bubble method that requires a steady‐state concentration of agent in the circulation, the droplet method can be used to create bubbles at the same location in, say, the arterial and portal phases of liver contrast, so effectively resolving two vessels within the beam by creating bubbles first in the hepatic artery and second in both the artery and portal vein.

Before it can be applied, several aspects of the droplet method remain to be investigated. First, it needs to be shown that the echoes from the bubbles created by vaporization report the tracer concentration. Figure 2 shows that over about an order of magnitude of droplet concentration, mean bubble echo intensity bears an approximately linear relationship to droplet concentration. It is noted that there are differences in the absolute value of intensity for different droplet batches, and this is likely to reflect intrinsic variations in the manual process of bubble condensation we use to synthesize the droplets: better controlled processes are clearly possible. However, the proposed method does not rely on reproducible levels of absolute enhancement from a given vial, only that it bears a linear relationship to concentration. Figure 4 shows that the in vitro half‐life of the activated bubbles is relatively short, at 2–3 seconds. This is probably related to the shell properties of the bubble, which have been shown to derive from the shell material of the droplets.^39^ While there are many reasons to require a longer half‐life in future in vivo applications, the present one is not amongst them, as the measurement of depletion of bubbles from the region of creation is very rapid, and in most cases is complete by 200 ms.

Figure 5 shows that using echo intensity integrated over the ROI as a measure of concentration, a measured flow percentage error of up to 35% was obtained, with a coefficient of variation under 10%. While these initial estimates are clearly in need of improvement, the accuracy and reproducibility are comparable to those of existing ultrasound techniques to measure flow using Doppler.^31^ An obvious source of error is in the uncertainty of the volume of the region of interest containing the microbubble bolus. The elevational beam width at the depth of the ROI must be known for an accurate estimate of the ROI size and its sensitivity function convolved with the distribution of echo intensity. Future work should consider the effect of the non‐uniform pressure field on droplet vaporization, much as has been done for microbubble disruption in the negative bolus technique.^1^

**Figure 7:**
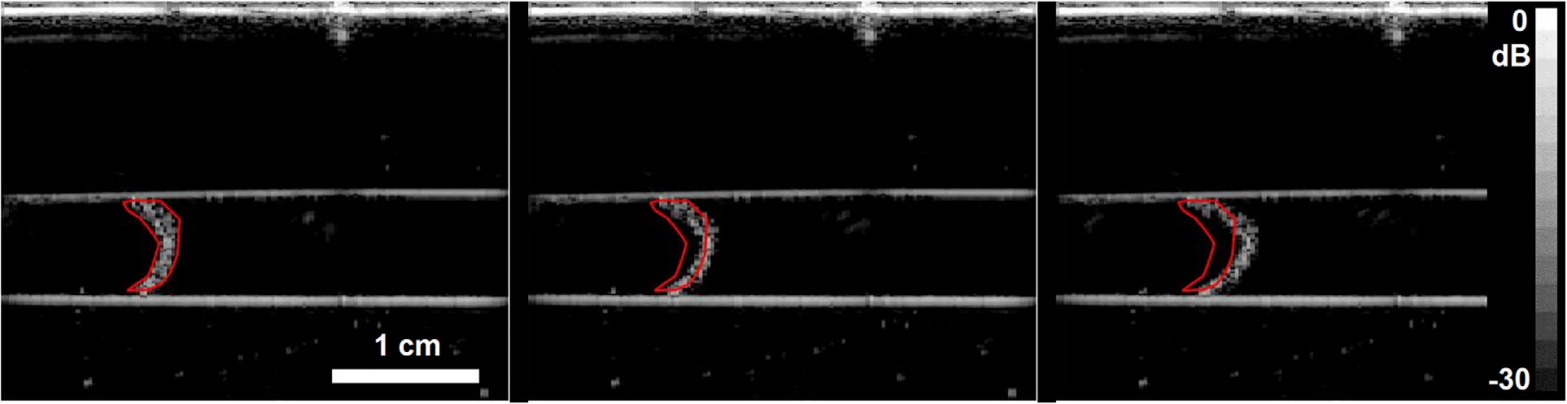
B‐mode image of a microbubble bolus with an automated region of interest (red) drawn around it. Left: 10 ms post activation; middle: 60 ms post activation; right: 120 ms post activation. Vessel diameter is 0.66 cm and the flow rate is 40 mL/min.

Figure 6 indicates that in this setting, the flow ratio between two adjacent vessels could be measured with an average percentage error of about 25%. However, the method of integrating echo intensity is not the only way in which tracer concentration can be estimated. Figure 7 shows that in this experiment, it was possible to resolve individual bubbles. By compressing the echo from each bubble into a binary level and counting bubble “events,” the measurement will be less prone to variation induced by attenuation and characteristics of the beam geometry, both of which have a strong effect on echo intensity. If feasible, counting bubble echoes may be a more robust method for estimating flow than relying on echo intensity, as has been reported in super resolution bubble imaging experiments^40^ and is commonly used for flow measurement with radioactive indicators.

A number of issues would require attention for the eventual use of the proposed method. First, in spite of the use of planewave imaging, a more rapid acquisition will yield more accurate results. The maximum velocity of the bubble cloud in this experiment was 14 cm/second, implying that it will have moved about 250 μm in the 1.8 ms needed to form each of the pulse inversion pairs. As the wavelength is 440 μm, decorrelation between pulses will occur that creates an artefactual signal in addition to the nonlinear echo (with pulse inversion, signal amplitude increases with motion decorrelation). Thus a more rapid acquisition, either by reducing the number of compound angles or increasing the PRF, would be advantageous. Both should be possible in future implementations. Second, as with the bubble disruption‐replenishment method for flow measurement,^1^, ^2^ account must be taken of the diffraction characteristics of the beam, in particular in the elevation direction, which changes with depth when using a 1‐dimensional array. In a potential clinical application for measurement of HPI, both the hepatic artery and portal vein lie in close association at the same depth, so while tissue attenuation may be equal for them both, the larger diameter of the portal vein will make it necessary to account for beam characteristics at the point of measurement. Further, errors due to partial volume insonation of the vessel would likely be greater at the elevational focus. While this method might be used to determine the HPI in the main hepatic artery and portal vein, creating a map to measure regional distribution of HPI within the liver would require the development of droplets that produce longer‐lasting microbubbles. Liver transit times as high as 65 seconds have been measured^41^; the DFB DBPC/DPPE‐PEG5K droplets used here would not have time to adequately perfuse the liver when vaporized at the *porta hepatis*.

## Conclusions

This in vitro study demonstrates the feasibility of using a positive bolus tracer, induced by image‐guided ultrasound excitation, to measure flow locally in deep vessels. The perfluorobutane sub‐micron droplets used here are promising candidates, as they can be converted to microbubbles using ultrasound exposures within the diagnostic range of exposure intensity. The ratio of flows between two vessels, ranging between 0.25 and 4, was measured by this method to a percentage error within 25%. Additional work is needed to make comparable measurements in a metered in vivo setting, and to develop phase‐change droplet agents that produce bubbles with a longer lifetime.

## Data Availability

The data that support the findings of this study are available from the corresponding author upon reasonable request.
